# The recovery and resilience plan on the long-term care system. Towards a deinstitutionalization?

**DOI:** 10.3389/fpubh.2023.1130132

**Published:** 2024-01-08

**Authors:** Fernando Bermejo-Patón, Raúl del Pozo-Rubio, María Elisa Amo-Saus, Pablo Moya-Martínez

**Affiliations:** ^1^Department of Economics and Finance, School of Social Sciences, Castilla-La Mancha University (UCLM), Cuenca, Spain; ^2^Department of Economics and Finance, School of Economics and Business Administration Castilla-La Mancha University (UCLM), Albacete, Spain

**Keywords:** long-term care, recovery and resilience plan, social accounting matrix, Input–Output methodology, economic return, deinstitutionalization

## Abstract

**Introduction:**

After the crisis caused by Covid-19, among other socioeconomic problems, the fragility of the organizations that make up the Spanish Long-Term Care System was revealed. These events prompted the Recovery and Resilience Plan (RRP). The aim of this study is to estimate the socioeconomic impact on Long-Term Care (LTC) of the investment delivered by the RRP. In addition, to fulfil our main aim, a secondary and necessary aim was to calculate the most current social accounting matrix (SAM) of the Spanish economy.

**Methods:**

We analyse the components of the demand linked to the RRP investment allocated to LTC, and subsequently, based on Input–Output methodology, we calculate a social accounting matrix (SAM) of the Spanish economy to estimate the overall economic return.

**Results:**

The results obtained using the SAM model proposed herein evidence the multiplier effect of the RRP invested in LTC. Every euro allocated to the RRP generates 4 euros in income for Households, Firms and the External Sector, 3.4 euros in industrial output, and returns 0.6 euros in taxes and social contributions to the Government. This also entails creating 26,410 direct and indirect jobs as well as 10,059 induced ones.

**Discussion:**

Given the severe recession scenario triggered by the consequences of COVID-19, the results of this study highlight the significant multiplier effect that RRP investment may generate to alleviate the downturn in the Spanish economy and, more specifically, in the Spanish LTC System.

## 1 Introduction

According to data provided by the World Health Organization (1), Covid-19 has caused more than 4 million deaths, with approximately 186 million people having been infected. Since the pandemic began, social distancing has been the fundamental strategy to stop the spread of the virus. This measure was the main cause of the lockdown in March and April 2020, which, together with the fear of contagion and the uncertainty of households and institutions, had a significant impact on economic activity and employment. In addition, this recession has occurred in a context of general low economic growth [secular stagnation according to Summers (2)], high levels of indebtedness, extreme inequality in the distribution of income, population aging in advanced economies (3) and serious hysteresis problems (4).

**Figure 1:**
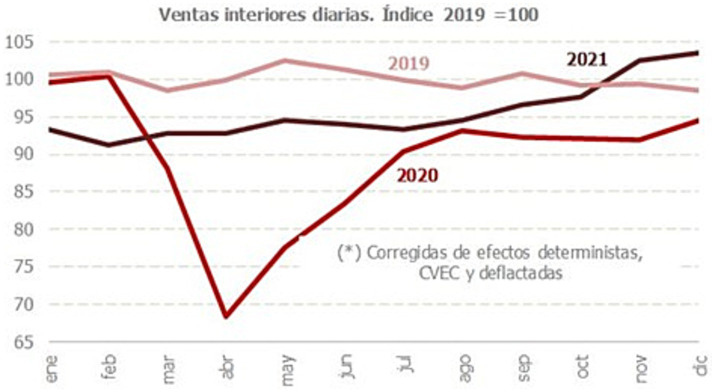
Trend of economic activity in Spain (2019–2021). Source: Agencia Tributaria (6).

The impact on the Spanish economy has been especially significant. According to IMF (5), the main reason is the importance of the tourism sector, in addition to the scarcity of large companies and the large number of temporary employment contracts. Figure 1 clearly shows the decline in economic activity in Spain during 2020 and part of 2021 compared to 2019, taking as a reference the sales data collected by the Spanish Tax Agency (6). These data reflect that the immediate consequence of the lockdown announced in March 2020 was an intense drop in sales, which decreased by more than 30% and which, in some activities, reached 100% over a long period. As can be seen in Figure 1, after the minimum reached in April 2020, a strong recovery process began in the first weeks, which stagnated in August, and then resurged in December when activity appeared to approach the initial level.

This economic context has made it necessary to implement a reform plan, not only to support the post-crisis recovery, but also to counteract the impact of this crisis on economic activity. In this respect, the instruments made available by the European Community to its Member States will play a decisive role. Among them, the Recovery and Resilience Facility is the main policy measure employed to mitigate the economic and social impact of the coronavirus pandemic and make European economies and societies more sustainable and resilient. As stated in European Commission (7), the Facility is a temporary recovery instrument that allows the Commission to raise funds to help each Member State implement reforms and investments through its national Recovery and Resilience Plan (RRP). To benefit from the support of the Facility, Member States must submit their RRP to the European Commission, setting out the reforms and investments to be implemented by end-2026. The Member States can then receive financing up to a previously agreed allocation.

This study focuses on evaluating the effect that the Recovery and Resilience Plan (RRP) may have on the demand and production associated with care under the Spanish Long-Term Care System (LTCS) (8). According to information published by the Government of Spain, the objective of the RRP is to accelerate economic and social recovery after the COVID-19 crisis and to increase growth capacity in the medium and long term. The RRP is organized in four cross-cutting pillars (ecological transition, digital transformation, territorial and social cohesion, and gender equality) that are aligned with the six basic pillars of the EU Recovery and Resilience Facility (7). As further explained in the Annex 1 of the Supplementary material those four pillars of Spain’s RPP are structured around 10 policy areas that define the bulk of investments in 30 components ranging from the urban agenda, the fight against depopulation and the development of agriculture, to the modernization and reinforcement of the tax and pension system. Other areas include the improvement of infrastructures and ecosystems, education, and the modernization of science and business.

Within the eighth policy area, the RRP seeks to promote well-being by improving care, in addition to reinforcing the three traditional pillars of the Welfare State (education, health and social services). To do this, it addresses the issues of financing and managing organizations and the social capital that the system brings together, efficiently articulating the powers of the different public administrations and public-private cooperation for the implementation of personal care, reinforcing mechanisms and equipment for long-term care, incorporating new technologies to improve home care and promoting universal accessibility.

The first specific challenge to be addressed is to promote change in the long-term care model, introducing reforms that simplify procedures and reduce waiting lists, reinforce the quality of professional services and conditions and increase the coverage of benefits. The key question is to promote services that reinforce more person-centred care and promote deinstitutionalization. The reinforcement of care contributes to the objectives of the demographic challenge in the areas affected by depopulation and is aligned with the actions related to older adults, active aging and care for dependency, which constitute one of the action lines of the National Strategy against the Demographic Challenge (9).

Additionally, the care sector has a high capacity for job creation, mainly as a result of the rise in life expectancy in Spain. These jobs are also non-polluting, non-relocatable and essential for enhancing the well-being of the population. There remains considerable scope for improvement in the demand for long-term care in Spain. The country invests only 0.75% of GDP in this care, which is half the OECD average. Investing in care will reduce the structural barriers that limit women’s access to the labour market, helping increase the female employment rate, generating important tax returns in the future and expanding the base of Social Security contributors.

Against this backdrop, the aim of this paper is to estimate the socioeconomic impact of improvements in organizational structure, social capital and well-being, financed by the investment derived from the RRP. Thus, we first set out to analyse the demand that will be generated in the Spanish economy with the application of the funds destined to improve long-term care. In a second step, we study its impact on production and, subsequently, on the generation of employment and income in Spain, breaking this down, to a large extent, by economic sectors.

To this end, we calculate the social accounting matrix (SAM) multipliers of the Spanish economy in 2021 to subsequently obtain the effect of the demand shock generated by RRP investment allocated to the LTCS.

This article is organized in four sections. Following this introduction, the second section describes the general characteristics of the RRP in Spain and the measures it includes in relation to dependent care. The third section focuses on the methodology employed and the data sources used, while the fourth section presents the preliminary results obtained, and draws conclusions.

## 2 Materials and methods

This analysis uses a SAM model based on Input–Output methodology to calculate the impact of the measures included in the RRP on the Spanish economy at a sectoral level. Initially, the Input–Output Tables (IOTs, hereinafter) developed by Leontief (10) were mainly focused on analyzing the effects produced in industrial activity by exogenous changes in final demand and by the exchange of goods and services between different economic sectors. The annex to this document contains a more detailed specification of the characteristics of the Leontief model. Briefly, the equilibrium solution in the model allows us to determine the increase or decrease in production at the sectoral level in response to changes in final demand. In our case, this refers to the investment derived from the RRP.

However, the results obtained with the basic Leontief model omit the effects caused by the interrelationship between production, production factors, income distribution and final demand. The SAMs represent an extension of the tables used in the Input–Output model with which the previous limitations are overcome. A SAM is a square matrix whose elements represent the transactions carried out in an economy over a specific period (11). The economic models based on the SAM allow for more efficient modelling of the relationships between added value and final demand to complete the circular flow of income.

SAMs based on Input–Output methodology are of great importance as a tool to estimate various socioeconomic spillover effects in a country’s economy and they have been widely used in research evaluating the impact of different demand shocks in a national economic system (12–20), and also at regional level for the case of Spain (21–24).

As indicated in Cardenete et al. (21), SAMs integrate data from the National Accounts into the basic Input–Output model, thus completing the interdependence of the productive sectors and final demand with the exchanges that take place between productive factors and final demand. At a general level, a SAM contains production accounts, income distribution accounts in which factors of production occur, income use accounts in which operations between institutional sectors appear, capital transactions, and accounts which include exchanges with the External Sector.

Table 1 shows the basic structure of a generic SAM, which reflects the circular flow of income for an economic system. Such a structure is applied in this analysis for the Spanish economy in 2021. The rows represent the income received from the elements of each column and the columns reflect the income distributed between the elements of each row. Therefore, each component of the SAM indicates the bilateral flow between the accounts that come together in that element, such that a cell *ij* of the SAM would correspond to the income of the sector of row *i* that comes from the sector of column *j*. Given that the SAM contains all the transactions carried out by the agents of the economy, the accounting identity by which the expenditure carried out by the economic agents must be equal to the income obtained must be fulfilled and, consequently, the sum of each column of the SAM must be equal to the sum of each row.

**Table 1:** Basic structure of the social accounting matrix.

	Sectors	Wages	Social contributions	Capital	Other net taxes on production	Indirect taxes on products	Direct taxation	Induced consumption of households	Government consumption	Endogenous investment	Imports	Exports	Exogenous consumption of households	Exogenous investment
Sectors	Interindustry consumption (64 × 64)							Consumption	Consumption	Investment		Exports	Consumption	Investment
Wages	Wages													
Social contributions	Social Contributions							Social Contributions				Social Contributions	Social Contributions	
Capital	GOS + RM													
Other net taxes on production	Taxes													
Indirect taxes on products	Taxes							Taxes	Taxes	Taxes			Taxes	Taxes
Direct taxation								Taxes	Taxes			Taxes	Taxes	
Induced consumption of households		Wages	Social contributions	ENE + RM				Consumption						
Government consumption			Social contributions		Taxes	Taxes	Taxes	Transfers				Transfers		
Endogenous investment				Fixed Capital consumption				Savings	Savings			Savings	Savings	
Imports	Imports	Wages	Social contributions		Taxes	Taxes	Taxes	Imports						
Exports														
Exogenous consumption of households									Transfers					
Exogenous investment				Inv				Savings	Savings			Savings	Savings	

Source: Own elaboration.

To better harmonize the SAM data with the final demand components obtained from the 2021 National Accounts records, the table used in the 2015 Input–Output models was projected to 2021, following the Euro method described in Eurostat (25). The fundamentals of this method involves an iterative procedure that, in this case, allowed us to make the estimates for 2021 using the projection from 2015 to 2021 of the value added at the sectorial level and of the various categories included in the final demand block, both contained in the National Accounting records prepared by the INE.

Equation 1 shows the equilibrium solution of our SAM model, which captures the effect of an exogenous demand shock $df^{PRR}$ on the economy in terms of total output $x^{PRR}$ by using the SAM multipliers matrix M, which is further explained in the Annex 2 of the Supplementary material.


(1) $$x^{PRR}=M\cdot df^{PRR}$$

$df^{PRR}$ is a column vector of size 29 × 1 containing the investment allocation for the specific economic sectors according to the RRP targets for LTC. Applying multipliers matrix M of size 29 × 29 to $df^{PRR}$ allows us to obtain the column vector of size 29 × 1 of total output $x^{PRR}$, not only as regards the initial investment requirements, but also including the spillover effects on the industrial and institutional sectors of the economy. Once $x^{PRR}$ is known, employment $l^{PRR}$ depending on this level of output can be obtained as follows:


(2) $$l^{PRR}={\hat{l}}_{d}\cdot x^{PRR}$$

where ${\hat{l}}_{d}$ is a diagonal matrix where $l_{d}=\frac{l_{j}}{X_{j}}$ is the vector of direct coefficients of employment l by industry.

**Figure 2:**
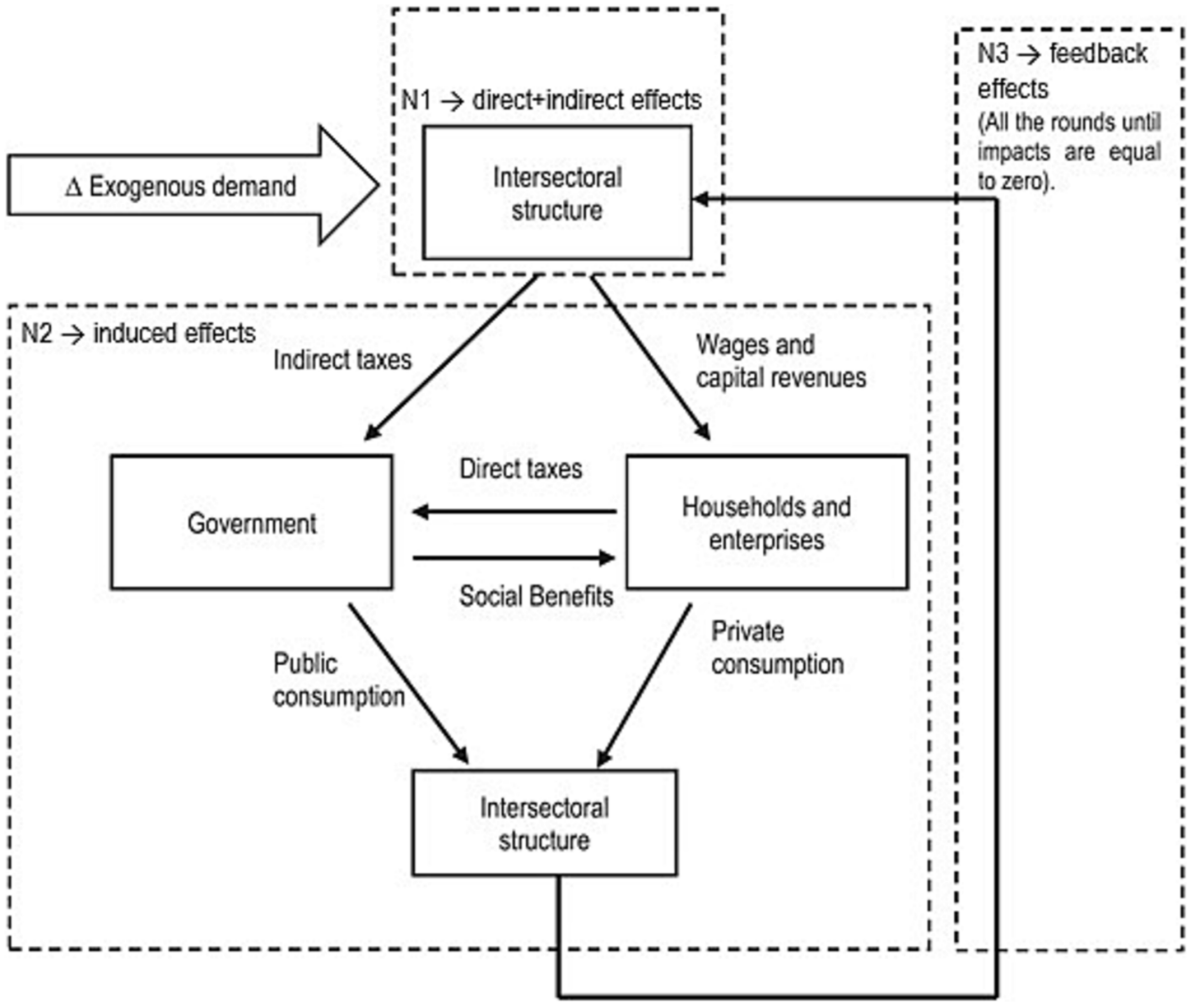
Decomposition of SAM multipliers. Source: Own elaboration based on (28).

This SAM model also allows us to split the total effect on output, employment and value added into what Stone (26) and Pyatt and Round (27) defined as N1, N2 and N3 multipliers, by partitioning the matrix in an additive fashion as follows:


(3) $$M=N_{1}+N_{2}+N_{3}$$

where N1 is the matrix of direct multipliers (or “own” multipliers), which includes only the traditional Leontief multipliers reflecting the monetary worth of sectoral output generated directly and indirectly to support the exogenous demand vector $df^{PRR}$. N2 is the matrix of indirect multipliers (or “open loop” multipliers), which records how the different components of exogenous demand vector $df^{PRR}$ are transmitted to households, firms and the Government. Finally, N3 is defined as the matrix of “closed loop” multipliers, capturing the feedback effects from households, firms and the Government and interindustry transactions. This additive decomposition, which is illustrated in Figure 2 and further explained in the Annex 2 of the Supplementary material, provides what Steenge et al. (29) called a “walk through the economic system”. This is fundamental to evaluate the share of output, income and employment depending directly on the demand shock caused by RRP investment and the induced and feedback effects resulting from the spillover impacts on the economic system.

## 3 Results

For the sake of clarity, the results obtained in this study are presented in three different sections. Firstly, the SAM results estimated for the Spanish economy in 2021 are presented; the multipliers resulting from the allocation of RRP investment are shown in the second section, and finally the results from the monetary flows are presented in the third section.

### 3.1 Social accounting matrix

This section presents the main characteristics of the SAM of the Spanish economy estimated for 2021. First, the different categories used to classify the industrial sectors, factors of production and institutional agents included in the SAM are described. In the case of productive sectors, the analysis was carried out considering the 64 categories that appear in the Statistical Classification of Economic Activities (NACE Rev.2). This classification is standardized and is compatible with the structure of the tables used in the Input–Output models for the Spanish economy prepared by the Spanish Institute of Statistics [INE, in its Spanish acronym; (30)]. For a better understanding of the results, the final sectorization scheme that will be applied in our SAM model follows the classification of 15 sectors considered by the INE, where the category of “Health Services and Social Services Activities” is divided into two independent activities, “Health care services” and “Social care services in residential establishments; social services without accommodation,” resulting in the classification of 16 sectors that appears in Table 2.

**Table 2:** Industry classification.

S01	Agriculture, forestry and fishing
S02	Energy supply, water supply and waste management activities
S03	Food, beverages, tobacco and textiles
S04	Manufacture
S05	Construction
S06	Wholesale and retail trade
S07	Transport
S08	Accommodation and food service activities
S09	Information and communication
S10	Financial and insurance activities
S11	Real estate activities
S12	Professional, scientific and technical activities; administrative and support service activities
S13	Public administration, defence and education
S14	Health services
S15	Social work activities
S16	Arts, entertainment and recreation; other service activities; activities of household and extra-territorial organizations and bodies

Source: Own elaboration based on the Input–Output framework for the Spanish economy (30).

The results in the submatrix of intersectoral intermediate flows in the SAM of dimension (16 × 16) are obtained by the Eurostat interactive method described in the methodology section. Having defined the block of accounts for flows between productive activities, the accounts related to the two productive factors considered (Labour and Capital), the Investment/Savings account and the accounts that represent the institutional sectors in the model are described below (Households, Public Sector, Financial and non-financial Institutions, External Sector). This information is contained in the SAM’s submatrix (13 × 16) of primary factors and in the submatrix (16 × 13) of final uses. The first 4 rows of the primary factor submatrix make up a fundamental block (4 × 16) that contains the added value components corresponding to the remuneration paid from the different productive sectors for the use of labour and capital factors (salaries, gross surplus exploitation and mixed income, social contributions and other net taxes on production). Reading this block by columns allows us to observe the distribution of remunerations to the different economic sectors for the use of productive factors, thus reflecting the process of primary distribution of income.

The rest of the elements in the submatrices of primary factors and final uses report on the flows of operations between the institutional agents of the model. Household activity is mainly reflected in the Consumption account, but also in monetary flows with the Public Administration and the External Sector in the form of transfers and taxes. In turn, the Public Sector is represented by its own current spending, social contributions paid by employers, social contributions received, net indirect taxes on production, taxes on products and imports and direct taxes (Personal Income Tax). Finally, the external sector is mainly represented by imports and exports, together with transfers from institutional sectors exchanged with the rest of the world.

For the purpose of this study, consumption operations are disaggregated into two accounts to distinguish between autonomous household consumption, which does not depend on the remuneration of production factors, and endogenous consumption, which is associated with the wages received by households for their participation in production. Applying a similar criterion, investment was broken down into an account that reflects endogenous gross capital formation linked to the increase in productive capacity and an exogenous account associated with autonomous investment. Similarly, the external sector was broken down into the endogenous part, corresponding to imports that depend on the income generated in production, and the exogenous part, which corresponds to exports that depend on the income generated in the rest of the world. The purpose of this distinction is to identify the endogenous part of consumption, gross capital formation and imports that will enter into the endogenization process linked to the calculation of the multipliers of the SAM model, as opposed to the exogenous component of demand, which is formed by the consumption of households covered with income that does not come from the production process, residential investment by households, plus investment in modernization of companies, and exports.

### 3.2 Impact of RRP investment in the Spanish long-term care system

This section presents the main results obtained from the SAM model described above. First, the sectoral disaggregation of the RRP investment focused on the LTCS is presented. Following the report of the 22^nd^ component addressing Spain’s RRP, the amount of investment proposed to enhance the LTC system is 2,083.9 million euros. These investment funds are mainly intended for the following purposes: evaluations and analyses; dissemination and awareness-raising campaigns; the construction and refurbishment of residential institutions; remodelling and equipping innovative day-care centres; technology for long-term care. As can be seen in the first column of Table 3, the full amount of 2,083 million euros can be split into certain components of a column vector that entails the exogenous demand shock to be estimated using our SAM model. The distribution by industry was implemented according to the information published in the 22^nd^ component report, where the total amount to be invested is split into different components as follows: 1,282.8 million euros to the economic sector of “Construction” for construction and refurbishment of residential institutions, and for the acquisition of equipment; 275.4 million euros to the economic sector of “Information and communication” for investment in technology for long-term care; 12.2 million euros to the economic sector of “Professional, scientific and technical activities; administrative and support service activities” to perform evaluations, analyses and dissemination and awareness-raising campaigns; 123.7 million euros to each of the economic sectors “Health services” (34.3 million euros) and “Social work activities” (89.4 million euros) for the remodelling of innovative day-care centres. The remaining amount of investment is included in the indirect taxes linked to these purchases (146.8 million euros) and in imports (242.9 million euros).

**Table 3:** PRR investment allocation and total output generated.

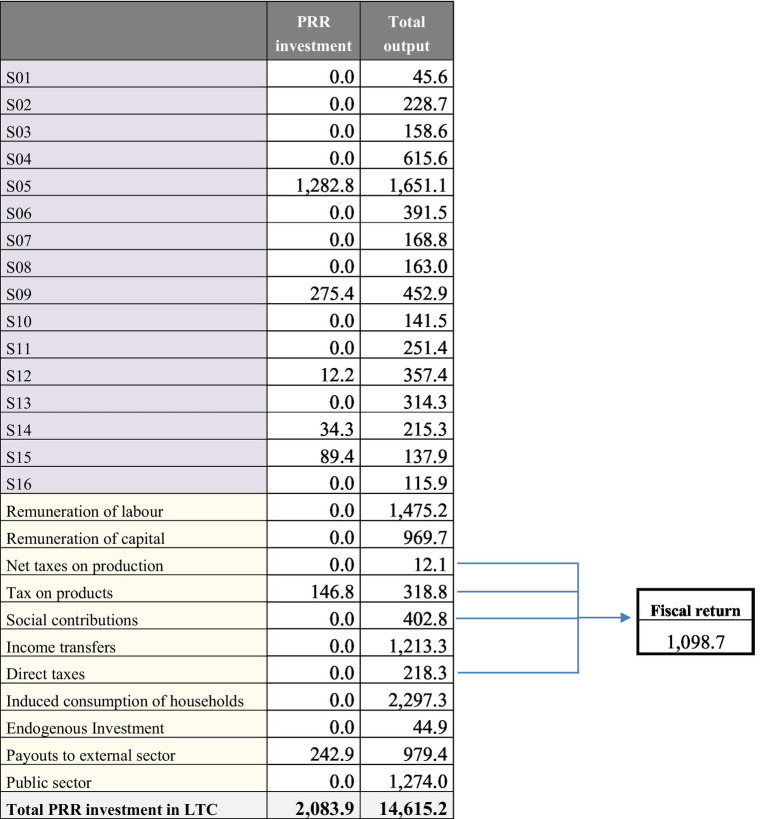

Results in million euros. S01: Agriculture, forestry and fishing; S02: Energy supply, water supply and waste management activities; S03: Food, beverages, tobacco and textiles; S04: Manufacture; S05: Construction; S06: Wholesale and retail trade; S07: Transport; S08: Accommodation and food service activities; S09: Information and communication; S010: Financial and insurance activities; S011: Real estate activities; S012: Professional, scientific and technical activities; administrative and support service activities; S013: Public administration, defence and education; S014: Health services; S015: Social work activities; S016: Arts, entertainment and recreation; other service activities; activities of household and extra-territorial organizations and bodies.

Source: Own elaboration.

The second column of Table 3 shows the results of the total output generated by the above-mentioned demand shock. This level of output is not only determined by the sectoral impact on production derived from the allocation of RRP investment previously described, which is considered the direct impact. To estimate the total gain in production, it is also necessary to take into account that the initial demand shocks caused by RRP investment are transmitted to the production system, where interindustry backward linkages generate a multiplicative effect on output, which is considered the indirect impact. Moreover, besides industrial inputs, economic sectors also demand additional labour, increasing the total wage amount in the economy that will subsequently be spent by households, generating a complementary round of demand shocks, which is considered the induced effect.

Consequently, the output results in Table 3 include the total effect derived from the initial demand shock caused by RRP investment (direct, indirect and induced effects). At the aggregate level, this means that 2,083.9 million euros of increased demand creates 5,409.5 million euros of total output not only in the investee sectors, but also within the rest of the economic system. Consequently, the monetary RRP investment effect accounts for an overall multiplier effect on production of 2.6 from the initial investment (2,083.9 million euros). The sectors “Construction” (30.52%) and “Manufacture” (11.38%) benefit most, followed by “Information and communication” (8.37%), “Wholesale and retail trade” (7.24%), “Professional, scientific and technical activities; administrative and support service activities” (6.61%), and “Public administration, defence and education” (5.81%).

Apart from these effects on production, the SAM model can contribute to evaluating the systemic impacts to understand and quantify the income generation from the RRP investment. As can also be seen in the second column of Table 3, the initial demand shock generates 1,475.2 million euros in wages and 969.7 million euros in gross operating surplus and mixed income to households and firms, while 402.8 million euros in social contributions and 12.1 million euros in other net taxes on production revert to the Government. This means that the total value added generated from the initial shock in demand accounts for 2,457 million euros, which leads to an overall multiplier effect on income of 1.2 from the initial investment (2,083.9 million euros). Furthermore, the total effect caused by RRP investment returns another 318.8 million euros to the Government in the form of indirect taxes on products and 218.3 million euros in direct taxes. It requires 44.9 million euros in additional investment and imports worth 979.4 million euros, and it allows households to increase income by 2,297.3 million euros, as well as transfers by the Government for 1,274 million euros.

**Figure 3:**
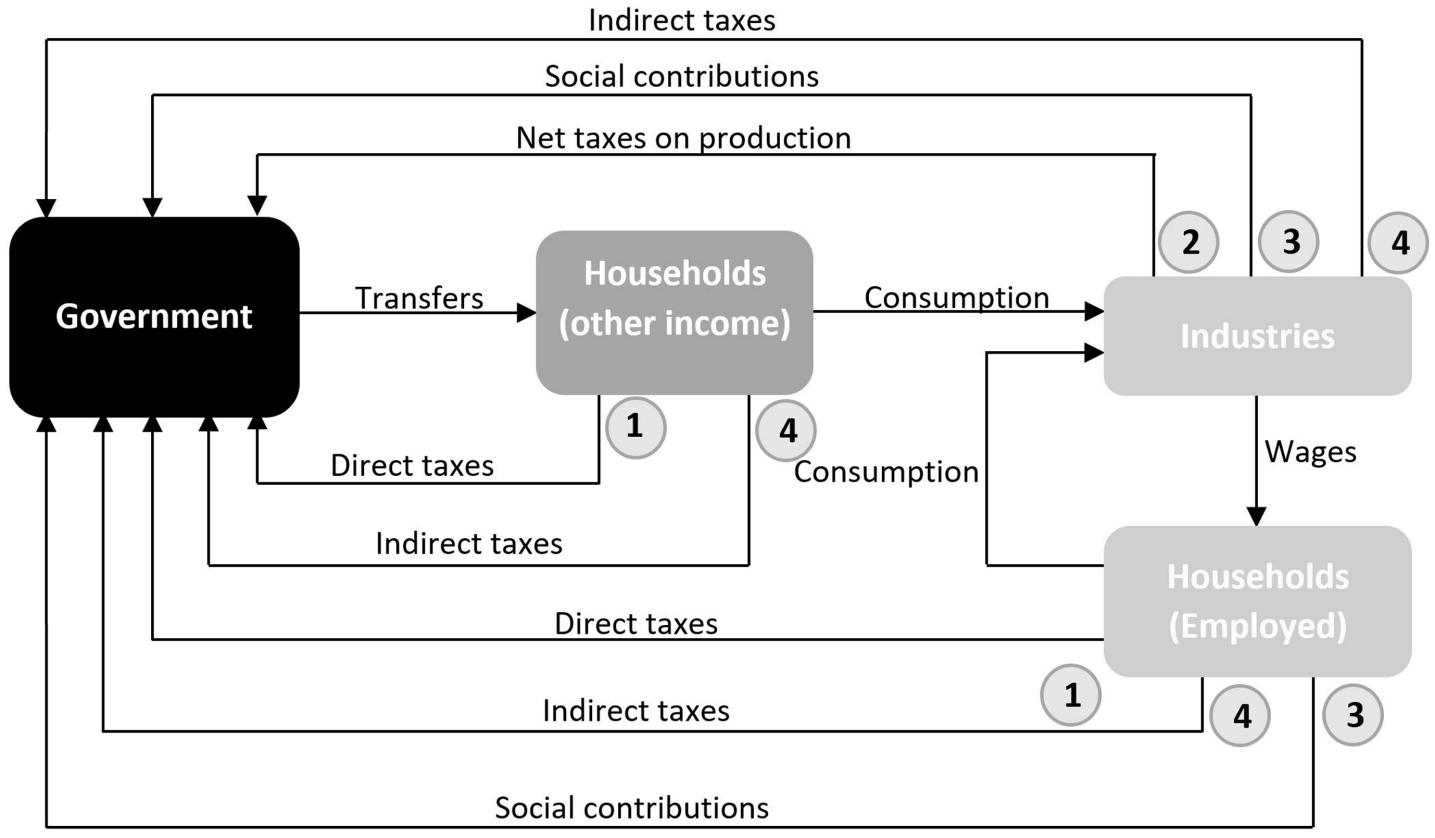
Fiscal return on PRR investment. Source: Own elaboration.

Thus, a large share of the income generated by RRP investment generates revenues for the Government via fiscal returns. Figure 3 shows the four possible channels through which the Government receives these revenues after the initial demand shock. The first would be the direct income taxes paid by households receiving transfers from Government and by households of workers involved in the production to meet RRP investment (218.3 million euros). The second channel involves the net taxes on production, derived from the increase in the level of output sustained by RRP investment (12.1 million euros). This increase in production includes all the direct, indirect and induced impacts previously defined. The third channel comprises the social contributions that employers and employees pay according to the total wages received by the workers depending on the RRP investment (252.4 million euros). Lastly, the fourth channel accounts for the indirect tax on goods and services purchased to meet the RRP investment (402.8 million euros). As Table 3 shows, the total fiscal return accounts for 1098.7 million euros, which is 52.7% of the initial amount invested.

The previous output results serve as the basis for calculating the number of jobs sustained by RRP investment according to Equation 2. Table 4 shows the employment by industry required to obtain the production generated by the initial demand shock. The first column contains total employment, while the remaining columns display the decomposition of the multiplier effect according to the three types considered in the study (direct plus indirect, induced and feedback). The results at the aggregate level reveal that 33.2% of the employment generated corresponds to direct jobs and 24.9% to indirect jobs, while induced jobs account for 22.1% and the remaining 19.8% is due to the feedback effect. Given that RRP investment is massively allocated to “Construction,” 27.9% of the employment generated is linked to this industry (12,688 jobs), followed by “Public administration, defence and education” (5,083.9) and “Professional, scientific and technical activities; administrative and support service activities” (4,742.1). The result in “Construction” is mainly based on the direct and indirect effect, which explains 96.3% of the employment generated in this industry (12,214.1 over 12,688 jobs) and accounts for 65.4% of the direct employment and 20.8% of the indirect employment sustained by RRP investment in all industries. Regarding induced employment, “Public administration, defence and education” is ranked first (3,632.9 jobs), followed by “Health services” (1,151.4) and “Wholesale and retail trade” (981.7).

**Table 4:** Employment sustained by PRR investment.

	Total effect	N1		N2	N3
		Direct effect	Indirect effect	Induced effect	Feedback effect
S01	504.6	0.0	87.7	134.3	282.6
S02	401.9	0.0	200.1	100.5	101.2
S03	505.2	0.0	132.7	121.7	250.8
S04	1,950.9	0.0	1,497.1	216.6	237.2
S05	12,688.0	9,857.8	2,356.3	234.4	239.5
S06	4,260.4	0.0	1,534.4	981.7	1,744.4
S07	1,169.9	0.0	528.4	283.1	358.3
S08	2,171.8	0.0	376.5	544.4	1,250.9
S09	3,812.6	2,750.0	793.7	138.1	130.9
S10	635.4	0.0	242.8	143.0	249.6
S11	459.8	0.0	236.4	84.3	139.1
S12	4,742.1	340.9	2,654.5	834.5	912.2
S13	5,083.9	0.0	259.3	3,632.9	1,191.7
S14	2,107.6	335.9	122.4	1,151.4	497.8
S15	2,768.8	1,795.0	60.9	605.4	307.4
S16	2,229.9	0.0	247.8	852.3	1,129.7
**Total**	**45,492.6**	**15,079.7**	**11,330.7**	**10,058.7**	**9.023,5**

Total effect = N1 Direct effect + N1 Indirect effect + N2 Induced effect + N3 Feedback effect. Employment in number of equivalent jobs. S01: Agriculture, forestry and fishing; S02: Energy supply, water supply and waste management activities; S03: Food, beverages, tobacco and textiles; S04: Manufacture; S05: Construction; S06: Wholesale and retail trade; S07: Transport; S08: Accommodation and food service activities; S09: Information and communication; S010: Financial and insurance activities; S011: Real estate activities; S012: Professional, scientific and technical activities; administrative and support service activities; S013: Public administration, defence and education; S014: Health services; S015: Social work activities; S016: Arts, entertainment and recreation; other service activities; activities of household and extra-territorial organizations and bodies.

Source: Own elaboration.

### 3.3 Final results from monetary flows

As described in Eq. 3 in the Annex 2 of the Supplementary material, the bottom right-hand element of the endogenous block of the SAM is the H matrix, which contains the monetary flows describing the secondary distribution of income from the Government to Households, Firms and the External Sector (on the receiving benefit side) and vice versa (on the contributing side, through the payment of direct income taxes, social contributions, taxes on products and net taxes on production).

The H matrix shows the systemic results of giving one unit of income to a particular institutional agent and its effects on the other institutional agents (Households, Firms, the Government and the External Sector in our analysis). The H matrix provides a summary of the results after a walk through the system, i.e., condensing the circular flow of income explained above. Reading the H matrix by columns reveals the multiplier effect of one unit of income on other institutional agents. By rows, it shows how much money is received by an institutional agent when increasing one unit of income in other institutional agents.

**Figure 4:**
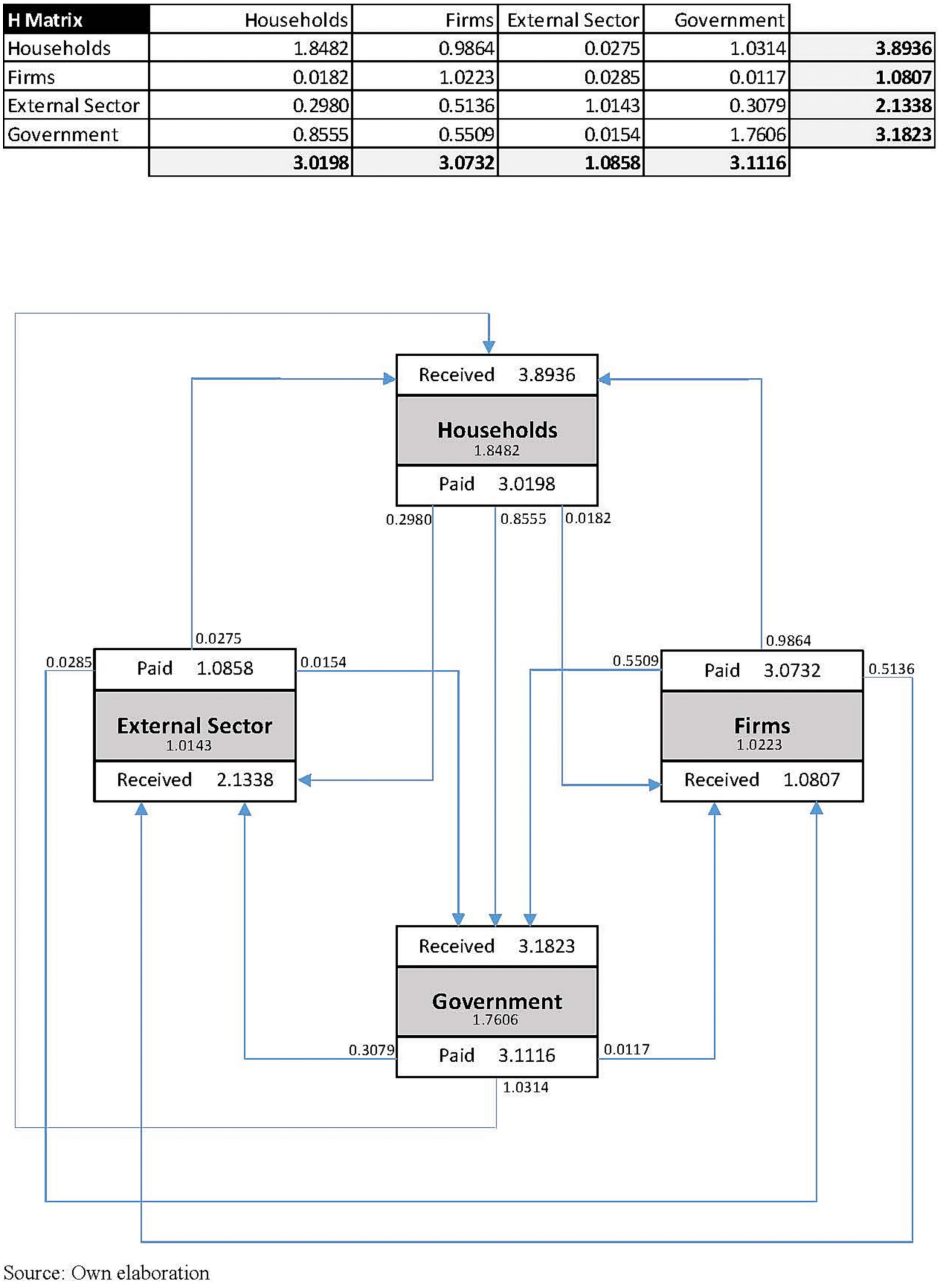
H Matrix and additive decomposition of multiplier effects. Source: Own elaboration.

Figure 4 gives an overview of the H matrix resulting from the demand shock caused by RRP investment. The Totals row reflects the multiplier effect for each euro received by every institutional agent. The flows below the H matrix show how the multiplier effect is generated for each institutional agent and subsequently paid to the other ones. Thus, the Government is the institutional agent generating the largest return with 3.1116 euros per euro received, which is further split into 0.3079 to the External Sector, 0.0117 to Firms, 1.0314 to Households and 1.7606 to the Government itself. The External Sector is the lowest income generator (1.0858 euros per euro received). The diagram also shows that Households are the institutional agent that benefits the most, earning 3.8936 euros per euro received by the other agents. Besides the above-mentioned 1.0314 euros received from the Government, Households receive 0.9864 euros from Firms and 0.0275 euros from the External Sector. In net terms, therefore, Households and the External Sector receive more than they pay (0.9719 euros and 1.0552 respectively), while the Government (−0.5237 euros) and particularly Firms (−1.9854 euros) show a negative balance.

## 4 Discussion

The emergence of the COVID pandemic at the beginning of 2020 was undoubtedly a turning point for the health and social fields, as well as for the other sectors of society. The economic accounts of countries deserve special attention. Public deficits increased by 11, 9.7, 9.5, 9.2, 7.2, 5.7 and 4.2% for Spain, Greece, Italy, France, the EMU, Portugal and Germany, respectively, with the corresponding increase in public debt to levels of 120, 205.6, 155.8, 115.7, 100, 133.6 and 69.8%, respectively (31). In response to this impact, the 30 lines of action corresponding to the RRP described in the introduction will be supported by the inflow of 140 billion euros in transfers and credits for the period 2021–2026 (12–14% GDP), which will enable a sustained increase in the Spanish GDP of 2.6 percentage points on average each year until 2031.

This is an ambitious plan for the Spanish economy that, if the design is properly executed, would produce a radical structural change in the functioning of the economy within the four main areas considered (ecological transition, digital transformation, territorial and social cohesion and gender equality) and their 10 levers. Success would allow Spain not only to rise in the ranking of developed economies, in terms of modernization, growth and development, but also to act as a leader in economic transformation, creating an itinerary that other world economies could replicate, addressing similar areas of action and applying comparable procedures. However, the evidence to date shows that funds are being received and distributed much more slowly than expected. This shortfall is exacerbated by the present commodity crisis, rising inflation and the increased cost of borrowing. Together, these factors make it very difficult to quantify the impact of the amount budgeted in the RRP.

The lines of action in the RRP referring to the Spanish LTCS focus on the transition towards a de-institutionalized model, featuring person-centred care and the development of community services and home care. Success in these areas would help overcome the limitations caused by the pandemic, and also correct inefficiencies in the LTCS that have accumulated since its inception, enabling managers to better address future challenges. Moreover, this approach takes social interests into account, as many individuals responsible for dependent persons are calling for a new model of care in which the latter can live most of the time at home, receiving the services they need and enjoying a higher quality of life and well-being (8).

The deinstitutionalization motivated by the fragility of the residential care system for older adults with the emergence of COVID-19, together with the premise that families should be able to remain at home for as much of their lives as possible, make it necessary to reorient the Spanish LTCS. Arguably, a feasible solution in the short term, and one that is plausible and viable in the long term, is the vigorous development of the home help service, together with a greater adaptation of homes to the needs of dependent persons. These changes should be effected placing the interests and preferences of dependents at the heart of the system. In the macroeconomic scenario, this would create a greater number of jobs (direct, indirect and induced) generate more added value and distribute income, lending more weight to social contributions, salaries and wages than to capital remuneration. However, we are currently unaware of the extent of these impacts, which depends on whether the service that generates them is residential care or home help. Accordingly, research is needed to estimate the impact inherent to each type of benefit and to assess the effect produced on the quality of life of dependent individuals (and by extension, that of their families). This focus is necessary so that policymakers can be offered accurate, appropriate information with which to determine the type of service that should be preferred.

In view of the above, this study establishes the bases to estimate the socioeconomic impact that the application of the RRP on the Spanish LTCS can produce in the Spanish economy. In the proposed procedure, we first describe the components of the demand linked to the investment initiatives included in the 22^nd^ component addressing Spain’s RRP and then estimate the total production necessary to satisfy this demand and calculate the jobs and income thereby generated, using our novel construction of the SAM for the Spanish economy.

The first outcomes presented focus on the features of the SAM, to evaluate the investment effects of the RRP. As previously stated, SAMs reflect the interdependence of the productive sectors and final demand with the exchanges that take place to distribute the level of income generated in the production process. This makes SAMs extremely useful for evaluating the economic impact of demand shocks, not only related to investment like the RRP plan presented here but also to spending on consumption by private and public institutions.

Having built the SAM, one of the first significant outcomes achieved, we then used it to model the RRP investment delivered in the Spanish LTCS. This modelling shows that an initial investment of 2,083.9 million euros creates 5,409.5 million euros of industrial output and generates 2,457 million euros in income for households, firms, government and the external sector, including a fiscal return of 1,098.7 million euros from taxes and social contributions. Additionally, the analysis shows that households benefit most from the RRP, receiving almost 4 euros from each euro generated, while the Government is the institutional agent obtaining the largest return (3.1 euros in one euro received under the Plan).

In addition, this level of production creates around 45.5 thousand jobs (33.2% of which are direct, 24.9% indirect, 22.1% induced and 19.8% derived from the feedback effect). From another standpoint, 2.18 jobs are created for every €100,000 invested. These results are certainly valuable as they prove that the type of investment provided by the RRP not only generates substantial benefits in terms of increasing tax revenues but also it supports employment, some of which is directly linked to the LTCS sector.

The Spanish LTCS was originally expected to have a very positive macroeconomic impact, creating 262,735 new jobs and 190,000 induced jobs, and potentially leading to the incorporation of 115,000 informal caregivers into the formal market. The expected annual fiscal return was around two billion euros, obtained as higher tax revenues and social contributions and a lower rate of unemployment (32). This forecast was subsequently re-examined. Thus, Herce et al. (33) calculated that fewer jobs would be created (in the range 160,000–175,000 jobs, depending on the methodology applied) Another study predicted a figure of 154,523 jobs, with an annual average of 137,000 jobs during the period 2007–2011, and a fiscal return of 27% via taxes and payroll contributions (34). A further study focused on the structural reform of the Spanish LTCS in 2012, concluding that it produced an additional 151,353 jobs in Spain (direct, indirect and induced) in 2012 (35).

However, to the best of our knowledge, only one study has evaluated the impact in Spain of the RRP, in terms of new jobs created. This study found that the provision of this extraordinary financing to the Spanish LTCS would generate 440,319 jobs (direct and indirect, plus induced effects) (28). This figure contrasts sharply with the 45,493 jobs estimated in the present study. The main reason for this discrepancy is that the cited study assumed public financing of 13,961 million euros, rather than the 2,083.9 million euros assigned officially in the RRP. Therefore, the present study is the first to estimate the macroeconomic impact of the RRP alone on employment.

The original design of the catalogue of benefits expected for the Spanish LTCS defines two large groups: service benefits and economic benefits (37). While the service ones have a significant preferential character, the economic ones have a residual and secondary character in preference of order of assignment. Previous studies have shown that the provision of services contributes to the creation of a greater number of jobs, production and added value than economic benefits. Thus, two out of every three jobs are generated by services while the remaining third are generated by economic benefits. If only service benefits were provided, in 2012, some 150,000 more jobs would have been created to meet the same needs of dependent people, replacing the economic benefits existing at that time (38). On the other hand, for every million euros invested in economic benefits, 16.88 jobs would be created (53.02% direct, 24.53% indirect and 22.45% induced), while every million euros invested in service benefits would generate 41.91 jobs of which 68.41% are direct, 9.16% indirect and 22.43% induced (35). In addition, given that obtaining these figures entails the use of Input–Output models, spillover effects on the different sectors of the economy can be observed. In another study, we showed the importance of the social work activities sector, given its weight within the dependency model, and its low return (39).

The main limitations of the present study are mostly related to the underlying assumptions of the Input–Output methodology. SAM models assume a fixed average technology for each economic sector, and so the input coefficients cannot change. In addition, the SAM model yields a constant return to scale and no supply constraints are considered, a question that is inadequately addressed in basic Input–Output models. Furthermore, the Spanish Input–Output table for 2021 has not yet been published, and so it was necessary to project the most recent Input–Output table available for Spain (2015), using National Accounting records. However, this approach might produce inaccuracies related to interindustry consumption.

## 5 Conclusion

The policy actions included in the RRP for the Spanish LTCS are intended to address the weaknesses in the system, both those pre-existing the pandemic and also, most especially, those that emerged during the crisis, specifically, the fragility of the residential care service (40). The emergence of COVID-19 in Spain led to the death of 26,500 dependent persons living in residential care, between March 2020 and May 2021, with an excess mortality of 43.5%, in addition to affecting mental health and quality of life, both for residents and family members (41).

The results of this study highlight the significant multiplier effect that RRP investment may produce to alleviate the downturn in the Spanish economy. To accomplish this, a SAM model was built for 2021, using data from basic IOTs and National Accounting data. SAM models are extremely efficient for describing how demand shocks generate and distribute production and income to both industrial and institutional sectors of the economy. Among other advantages of SAM models, they make it possible to estimate the impact on employment according to the level of production estimated and explain how institutional sectors benefit from income generation (by using the H Matrix embedded in the SAM).

The present study is of great methodological importance, providing a solid basis for evaluating different impacts on the economy (for example, we model the impact of increased or reduced expenditure on items such as pensions, education or other social priorities), by constructing a social accounting matrix (the most recent of its type). Finally, this new tool is used to evaluate the impact of the RRP, showing it to have very positive consequences for society. Nevertheless, further research is needed to provide a basis for upcoming analysis in enabling a comparison on how RRP investment has been allocated to healthcare (particularly to LTCS) and whether the potential return has met the initial expectations. The outcomes of this future research would not be only exceptionally interesting for Spain but also for the rest of European Member States.

## Data availability statement

Publicly available datasets were analyzed in this study. This data can be found at: https://www.ine.es/dyngs/INEbase/es/operacion.htm?c=Estadistica_C&cid=1254736176806&menu=resultados&idp=1254735976608#!tabs-1254736195147.

## Author contributions

FB-P, RP-R, MEA-S and PM-M: conceptualization, writing—review and editing, visualization, and supervision. FB-P: methodology and writing—original draft preparation. RP-R and PM-M: formal analysis, project administration, and funding acquisition. All authors contributed to the article and approved the submitted version.
