# Supplementary material for: The recovery and resilience plan on the long-term care system. Towards a deinstitutionalization?
Source: Front Public Health. 2024 Jan 8;11:1130132. doi: 10.3389/fpubh.2023.1130132 (PMC10800364; doi:10.3389/fpubh.2023.1130132)
Supplement: Supplementary file 1 [file Table_1.DOCX]

Supplementary Material

**Long-Term Care System after COVID-19. The socioeconomic impact of Recovery and Resilience Plan in Spain**

**Annex 1**

The ten policy areas or lever policies (L) included in the Recovery and Resilience Plan for Spain incorporate thirty components (C) or lines of action as follows:

L1: Urban and rural agenda, the fight against depopulation, and agricultural development

C1: Action plan to ensure sustainable, safe and connected mobility in urban and metropolitan areas

C2: Housing refurbishment and urban renewal plan

C3: Environmental and digital transformation of the agri-food and fisheries system

L2: Resilient infrastructures and ecosystems

C4: Conservation and restoration of ecosystems and their biodiversity

C5: Preservation of the coast and water resources

C6: Sustainable, safe and connected mobility

L3: Fair, inclusive energy transition

C7: Deployment and integration of renewable energies

C8: Electrical infrastructures, promotion of smart networks and deployment of flexibility and storage

C9: Roadmap for renewable hydrogen and its sectoral integration

C10: A fair transition strategy

L4: Public administration for the 21st century

C11: Modernization of public administrations

L5: Modernization and digitization of the industrial and SME fabric, recovery of tourism and promotion of Spain as an entrepreneurial nation

C12: Spain 2030 Industrial policy

C13: Foster the growth of SMEs

C14: Plan for the modernization and competitiveness of the tourism sector

C15: Digital connectivity, the promotion of cybersecurity and deployment of 5G

L6: Pledge for science and innovation, and Strengthening the capabilities of the National Health System

C16: National strategy for artificial intelligence

C17: Institutional reform and strengthening the capacities of the national science, technology and innovation system

C18: Renewal and expansion of the capacities of the National Health System

L7: Education and knowledge, lifelong learning and capacity building

C19: National Plan for Digital Skills

C20: Strategic plan to promote vocational training

C21: Modernization and digitization of the educational system, including early childhood education from 0 to 3 years)

L8: The new care economy and employment policies

C22: Emergency plan for the care economy and strengthening inclusion policies

C23: New public policies for a dynamic, resilient and inclusive labour market

L9: Promotion of the culture and sports industries

C24: Revaluation of the cultural sector

C25: Spain, an audiovisual hub of Europe (Spain AVS Hub))

L10: Modernization of the tax system for inclusive and sustainable growth

C26: Development of the sports sector

C27: Measures and actions to prevent and combat tax fraud

C28: Adaptation of the tax system to the reality of the 21st century

C29: Improving the effectiveness of public spending

C30: Long-term sustainability of the public pension system under the Toledo Pact).

**Annex 2**

Following the traditional definition in Miller & Blair (2009), the equilibrium of a basic Input-Output model can be represented in matrix form by the following expression:

$\mathbf{x}={(\mathbf{I}-\mathbf{A})}^{-1}\cdot\mathbf{df}$ (1)

Where $\mathbf{x}$ is total production by industry (*n x 1*), ${(\mathbf{I}-\mathbf{A})}^{-1}$ is the inverse Leontief matrix (*n x n*) in which $\mathbf{A}=\left\{ \mathbf{a}_{\mathrm{ij}} \right\}$ represents the technical production coefficients, whose elements contain the quantity of goods and services from sector i that are necessary to produce a unit of output from sector j, and $\mathbf{df}$ is the final demand vector (*n x 1*) of the economic system. Since the basic IO models focus solely on the relationship between final demand and production, SAMs also allow the mechanisms by which the production process determines final demand to be described.

The data contained in the SAM make available the design of economic models from which, among other results, multipliers can be obtained. These multipliers are defined as the coefficients of input needs per unit produced of each good. The exchange matrix in a SAM would be defined as follows (Miller & Blair, 2009).

$\bar{\mathbf{G}}\mathbf{=}\left( \begin{matrix} \bar{\mathbf{Z}} & \mathbf{F} \\ \mathbf{W} & \mathbf{B} \end{matrix} \right)$ (2)

Where the different economic relations are split into an endogenous part $\bar{\mathbf{Z}}$ and three components included in the exogenous final demand matrix $\mathbf{F}$, the exogenously generated income matrix $\mathbf{W}$, and the matrix $\mathbf{B}$ of exogenous income distribution.

If we focus on the endogenous part $\bar{\mathbf{Z}}$ it is necessary to distinguish between the intermediate flows between sectors, the transactions of the sectors included in the final demand, and the different components of value added:

$\bar{\mathbf{Z}}\mathbf{=}\left( \begin{matrix} \mathbf{Z} & \mathbf{0} & \bar{\mathbf{C}} \\ \bar{\mathbf{V}} & \mathbf{0} & \mathbf{0} \\ \mathbf{0} & \bar{\mathbf{Y}} & \bar{\mathbf{H}} \end{matrix} \right)$ (3)

where $\mathbf{Z}$ contains intersectoral intermediate flows, $\bar{\mathbf{C}}$ is the matrix involving those transactions related to endogenous consumption, $\bar{\mathbf{V}}$ represents the matrix of primary inputs or value added, $\bar{\mathbf{Y}}$ is the matrix that stands for the income distribution of the different categories of value added, and finally $\bar{\mathbf{H}}$ is the matrix showing the distribution of income from the institutional sectors to the different components of final demand.

Once matrix$\bar{\mathbf{Z}}$ is defined, the SAM multipliers could be obtained by the expression:

$\mathbf{S}\mathbf{=}\mathbf{Z}\boldsymbol{\cdot}{\hat{\bar{\mathbf{x}}}}^{\mathbf{-}\mathbf{1}}\boldsymbol{\to}\bar{\mathbf{S}}\mathbf{=}\left( \begin{matrix} \mathbf{A} & \mathbf{0} & \mathbf{C} \\ \mathbf{V} & \mathbf{0} & \mathbf{0} \\ \mathbf{0} & \mathbf{Y} & \mathbf{H} \end{matrix} \right)$ (4)

Where $\mathbf{A}$ is the technical coefficient matrix, $\mathbf{C}$ contains the coefficients of final consumption, $\mathbf{V}$ is the matrix of direct value-added coefficients, $\mathbf{Y}$ contains the income distribution coefficients to the institutional sectors, and 𝐇 is the matrix of income distribution coefficients from the institutional sectors to the different components of final demand.

Consequently, as indicated in Pyatt (1988, 2001) and Pyatt & Round, (1979), a clear analogy with the equilibrium of the basic IO model indicated in equation (1) can be made, giving the solution of the basic SAM model as follows:

$\bar{\mathbf{x}}\mathbf{=}\left( \mathbf{I}\mathbf{-}\mathbf{S} \right)^{\mathbf{-}\mathbf{1}}\boldsymbol{\cdot}\bar{\mathbf{df}}$ (5)

Where $\bar{\mathbf{x}}\mathbf{=}\left( \begin{matrix} \mathbf{x} \\ \mathbf{v} \\ \mathbf{y} \end{matrix} \right)$ is a vector that is made up of three subvectors: the vector $\mathbf{x}$ of total sectorial production, the vector $\mathbf{v}$ of total value added and the vector $\mathbf{y}$ of institutional sectors total income. On the right-hand side of the equation, $\bar{\mathbf{df}}\mathbf{=}\left( \begin{matrix} \mathbf{df} \\ \mathbf{w} \\ \mathbf{h} \end{matrix} \right)$ contains three subvectors: the exogenous demand vector $\mathbf{df}$, the vector of exogenous value added $\mathbf{w}$ and the vector of exogenous household income $\mathbf{h}$.

The SAM model also allows to examine deeper the relationships and links between the different components of the matrix $\left( \mathbf{I-S} \right)^{\mathbf{-1}}$. To disentangle these economic relationships, we can decompose the SAM multipliers in an additive fashion following Miller and Blair (2009). Starting by dividing the $S$ matrix into two additive matrices $Q$ and $R$:

$\mathbf{S}=\mathbf{Q}+\mathbf{R}; \mathbf{Q}=\left( \begin{matrix} \mathbf{A} & \mathbf{0} & \mathbf{0} \\ \mathbf{0} & \mathbf{0} & \mathbf{0} \\ \mathbf{0} & \mathbf{0} & \mathbf{H} \end{matrix} \right); \mathbf{R}=\left( \begin{matrix} \mathbf{0} & \mathbf{0} & \mathbf{C} \\ \mathbf{V} & \mathbf{0} & \mathbf{0} \\ \mathbf{0} & \mathbf{Y} & \mathbf{0} \end{matrix} \right)$ (6)

Then, we define $\mathbf{T}$ as:

$\mathbf{T}={(\mathbf{I}-\mathbf{Q})}^{-\mathbf{1}}\mathbf{R}$ (7)

And if we call $\mathbf{M}=\left( \mathbf{I}-\mathbf{S} \right)^{-\mathbf{1}}$ in a multiplicative way:

$\mathbf{M=}\left( \mathbf{I-S} \right)^{\mathbf{-1}}\mathbf{=}\mathbf{M}_{\mathbf{3}}\mathbf{M}_{\mathbf{2}}\mathbf{M}_{\mathbf{1}}$ (8)

Where:

$\mathbf{M}_{\mathbf{1}}={(\mathbf{I}-\mathbf{Q})}^{-\mathbf{1}}$ (9)

$\mathbf{M}_{\mathbf{2}}=\left( \mathbf{I}+\mathbf{T}+\mathbf{T}^{\mathbf{2}} \right)$ (10)

$\mathbf{M}_{\mathbf{3}}={(\mathbf{I}-\mathbf{T}^{\mathbf{3}})}^{-\mathbf{1}}$ (11)

The additive partitioning uses the $M1$, $M2$ and $M3$ decomposition to transform it into what ([Stone, 1984](#_ENREF_10)) and ([Pyatt & Round, 1985](#_ENREF_8)) defined as $N1$, $N2$ and $N3$ multipliers:

$\mathbf{M}=\left( \mathbf{I}-\mathbf{S} \right)^{-\mathbf{1}}=\mathbf{N}_{\mathbf{1}}+\mathbf{N}_{\mathbf{2}}+\mathbf{N}_{\mathbf{3}}=\mathbf{M}_{\mathbf{3}}\mathbf{M}_{\mathbf{2}}\mathbf{M}_{\mathbf{1}}$ (12)

Where $\mathbf{N1}$ is defined as the matrix of direct multipliers (or “own” multipliers), including only the traditional Leontief multipliers. This reflects the monetary worth of sectoral output generated directly and indirectly to support the exogenous demand vector $\mathbf{df}$:

$\mathbf{N}_{\mathbf{1}}=\mathbf{M}_{\mathbf{1}}={(\mathbf{I}-\mathbf{Q})}^{-\mathbf{1}}$ (13)

$\mathbf{N2}$ is the matrix of indirect multipliers (or “open loop” multipliers), which records how the different components of exogenous demand vector $\mathbf{df}$ are transmitted to the households, firms and Government:

$\mathbf{N}_{\mathbf{2}}=\mathbf{M}_{\mathbf{2}}\mathbf{M}_{\mathbf{3}}\mathbf{M}_{\mathbf{1}}-\mathbf{M}_{\mathbf{3}}\mathbf{M}_{\mathbf{1}}$ (14)

Finally, $\mathbf{N3}$ will be defined as the matrix of “closed loop” multipliers, capturing the feedback effects from households, firms and Government and interindustry transactions:

$\mathbf{N}_{\mathbf{3}}=\mathbf{M}_{\mathbf{3}}\mathbf{M}_{\mathbf{1}}-\mathbf{M}_{\mathbf{1}}$ (15)

**Bibliography**

Agencia Tributaria. (2022). Informe anual de Recaudación Tributaria. 2021 Retrieved from <https://sede.agenciatributaria.gob.es/Sede/datosabiertos/catalogo/hacienda/Informes_anuales_de_Recaudacion_Tributaria.shtml>

Eurostat. (2014). Eurostat Manual of Supply, Use and Input-Output Tables. *Office for Official Publications of the European Communities, Luxemburg*.

INE. (2016). Contabilidad nacional anual de España: tablas Input-Output. Resultados. Retrieved from <https://www.ine.es/dyngs/INEbase/es/operacion.htm?c=Estadistica_C&cid=1254736177058&menu=resultados&idp=1254735576581>

Miller, R. E., & Blair, P. D. (2009). *Input-output analysis: foundations and extensions*: Cambridge University Press.

Pyatt, G. (1988). A SAM approach to modeling. *Journal of Policy Modeling, 10*(3), 327-352. doi:<http://dx.doi.org/10.1016/0161-8938(88)90026-9>

Pyatt, G. (2001). Some Early Multiplier Models of the Relationship between Income Distribution and Production Structure. *Economic Systems Research, 13*(2), 139-163. doi:10.1080/09537320120052434

Pyatt, G., & Round, J. I. (1979). Accounting and Fixed Price Multipliers in a Social Accounting Matrix Framework. *The Economic Journal, 89*(356), 850-873. doi:10.2307/2231503

Pyatt, G., & Round, J. I. (1985). Regional accounts in a SAM framework. *Social Accounting Matrices: A Basis for Planning, World Bank, Washington*.

Steenge, A. E., Carrascal, A., & Serrano, M. (2020). Income distributions in multi-sector analysis; Miyazawa’s fundamental equation of income formation revisited. *Structural Change and Economic Dynamics, 53*, 377-387.

Stone, R. (1984). *Where are we now? A short account of the development of input-output studies and their present trends.* Paper presented at the Proceedings of the Seventh International Conference on Input-output Techniques (UN, New York).
